# Revision of the genus *Trypogeus* Lacordaire, 1869 (Cerambycidae, Dorcasominae)

**DOI:** 10.3897/zookeys.502.9049

**Published:** 2015-05-04

**Authors:** Eduard Vives

**Affiliations:** 1 Facultat de Biologia, Departament de Biologia Animal, Avda. Diagonal, 643, 08028 Barcelona, Spain

**Keywords:** Taxonomy, revision genus *Trypogeus*, Dorcasominae, southeast Asia

## Abstract

The ten species of the genus *Trypogeus* Lacordaire are revised. *Trypogeus apicalis* Fisher, 1936, is proposed as a new synonym of *Trypogeus javanicus* Aurivillius, 1925. A neotype for *Trypogeus sericeus* (Gressitt, 1951) and lectotypes for *Toxotus fuscus* Nonfried, 1894 and *Trypogeus javanicus* are designated. *Trypogeus fuscus* auct. nec Nonfried is a misidentification of *Philus ophthalmicus* Pascoe. All the species are described and keys are given for distinguishing the species. Photographs of the types of all the *Trypogeus* species are published for the first time.

## Introduction

Lacordaire (1868) described the Group IX *Dorcasomides*, amongst the representatives of the subfamily Cerambycinae, in “*légion II des Cérambycides vrais, Cohorte I, Cérambycides silvains, section-B, avec les yeux finement granulés*” and in this group he included the genera *Dorcasomus* Serville, 1834 and *Megacoleus* Lacordaire, 1869.

This division was elevated to Tribe Dorcasomini by Aurivillius (1912), who included six genera (*Dorcasomus* Lacordaire, 1869, *Neoclosterus* Heller, 1899; *Plectogaster* Waterhouse, 1881; *Aphelogaster* Kolbe, 1897; *Gahania* Distand, 1907; and *Lycosomus* Aurivillius, 1903). Quentin and Villiers (1970) separated the latter five genera into other tribes, leaving the genus *Dorcasomus* as the sole representative of the tribe.

Danilevsky (1979) proposed the status of subfamily Apatophyseinae for Lacordaire’s (1869) Apatophysides, originally comprising the genera *Apatophysis* Chevrolat, 1860; *Pachyticon* Thomson, 1857 and *Trypogeus* Lacordaire, 1869. Later, however, Özdikmen (2008), in agreement with the comments of P. Svacha and M. Danilevsky (1987, 1989), established the synonymy of the subfamilies Dorcasominae and Apatophyseinae.

## Material and methods

### Terminology and descriptions

Terminology for body parts and terminalia used in the text is explained in the general description below. General terminology follows Yamasako and Ohbayashi (2011) and Svacha and Lawrence (2014). The description of species and the key for identification focus mainly on the colour and the patterns of antennae, pronotum, elytra and legs.

Acronyms of the collections studied and mentioned in this work:

BPBM Bernice P. Bishop Museum, Honolulu, USA

DEIM Deutsche Entomologische Institute, Müncheberge, Germany

EVC Eduard Vives Collection, Terrassa, Spain

IRSNB Institute Royal des Sciences Naturelles de Bélgique

IZAS Institute of Zoology, Chinese Academy of Sciences, Beijing, China

KMC Kiyoshi Matsuda Collection, Takarazuka City, Japan

MNHN Muséum National d’Histoire Naturelle, Paris, France

NHML Natural History Museum, London, Great Britain

NRSC Naturhistoriska Riksmuseet, Stockholm, Sweden

NOC Nobuo Ohbayashi Collection, Miura City, Japan.

SMC Sergey Murzin Collection, Moscow, Russia.

USNM United States National Museum, Smithsonian Institution, Washington, DC, USA.

## Taxonomy

### Subfamily Dorcasominae

Taxon classificationAnimaliaColeopteraCerambycidae

Lacordaire, 1869

Dorcasomides Lacordaire, 1868: 456.
Apatophysides Lacordaire, 1869: 234.
Apatophysinae Danilevsky, 1979: (misspelling).
Apatophyseinae : Lobanov et al. 1981; Löbl and Smetana 2010.
Dorcasominae : Özdikmen 2008; Villiers et al. 2011; Vives and Heffern 2012; Svacha and Lawrence 2014: 34.

#### Note.

The subfamily Dorcasominae currently has 321 species in Africa, Europe, and southeastern Asia. Among these, 257 are endemic to Madagascar and the Comoros islands, 24 species are Oriental (Vives and Heffern 2006), 22 species are South Palearctic and 12 are Afro-tropical. Dorcasominae are not present in the western hemisphere, Australia or Polynesia (Villiers et al. 2011).

### Genus Trypogeus

Taxon classificationAnimaliaColeopteraCerambycidae

Lacordaire

Trypogeus Lacordaire, 1869: 236.
Toxotus (auct. nec Dejean, 1821).
Trypogeus Aurivillius, 1912; Boppe 1921; Hayashi and Villiers 1985; Chiang and Chen 2001; Vives 2007; Miroshnikov 2014.
Paranthophylax Gressitt, 1951: 50.
Paranthophylax Gressitt & Rondon, 1971.

#### Type species.

*Trypogeus albicornis* Lacordaire (by monotypy)

#### Description.

**Figures 1–8.**
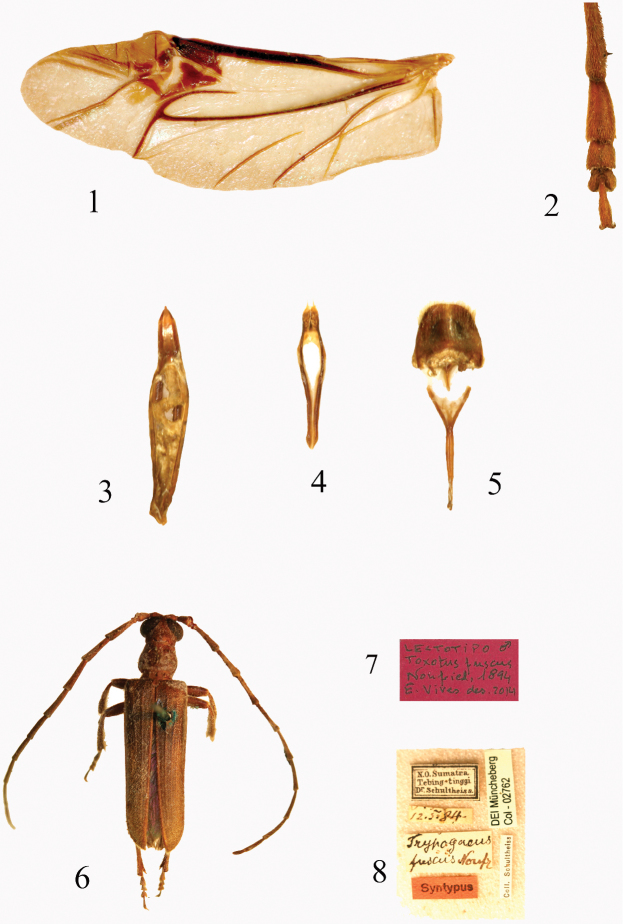
**1** *Trypogeus barclayi* Vives, left wing **2** *Trypogeus sericeus* female, tarsi from posterior leg **3** *Trypogeus gressitti* Miroshnikov, dorsal view of aedeagus **4** tegmen **5** pygidium and *spiculum gastrale* **6** *Philus ophthalmicus* Pascoe, (=Lectotype of *Toxotus fuscus* Nonfried) **7**–**8** *Trypogeus fuscus* labels of lectotype (DEI).

**Figures 9–10.**
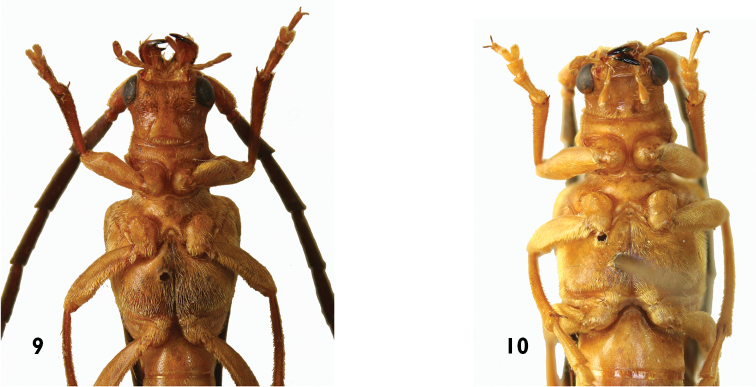
**9** *Trypogeus cabigasi* Vives, male underside **10** *Trypogeus barclayi* Vives, female underside.

**Figures 11–14.**
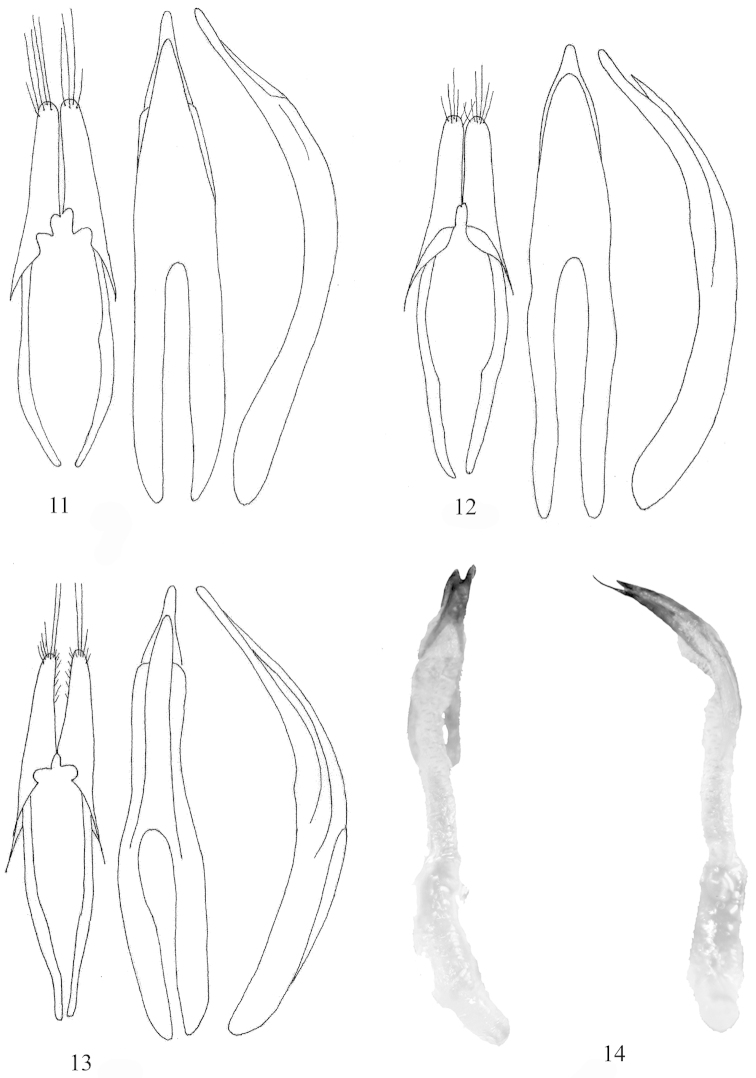
Tegmen, dorsal and lateral view of aedeagus **11** *Trypogeus cabigasi* Vives **12** *Trypogeus albicornis* Lacordaire **13** *Trypogeus barclayi* Vives **14** *Trypogeus coarctatus* Holzschuh, dorsal and lateral view of aedeagus and endophallus (drawings by Dr. N. Ohbayashi).

Anterior part of head with short rostrum. Long protruding mandibles, curved at the apex, the inner margin of the left mandible is sinuate and has no tooth whereas the margin of the right mandible is straight and has a sharp tooth in the middle. The neck is not narrowed or convex behind the eyes. Very long maxillary palpi, extending past the apex of the mandibles. Eyes moderately coarsely faceted; small and not very prominent. Antennae reaching or surpassing the apex of elytra in males, shorter in the females, the insertion is in front of the eyes as in other tribes of the Dorcasominae. The pronotum is subcylindrical, slightly wider than long at the level of the lateral protuberances, transverse in females, the anterior border is thin and simple and the posterior border is sinuate and margined. Discal area finely punctuate and presenting four or five gibbosities. Very short broad prosternum, the intercoxal process very narrow and dilated posteriorly. Procoxal cavities round and well extended towards the sides, almost reaching the lateral protuberance of the pronotum. Front coxae conical and protruding. Sides of the pronotum with a slight border in front of the protuberances. Mesocoxae closer together in males (Fig. 9), and more broadly separated by the mesosternal process in the females (Fig. 10). Triangular scutellum with rounded apex. Elytra not very long, flattened and strongly narrowed in the middle, slightly dehiscent at apex. In the females the elytra do not usually cover the abdomen and leave some terminal abdominal segments uncovered, particularly when it is swollen with eggs. The epipleura are well delimited and flattened. Wings (Fig. 1) are present and well developed in both males and females; they are translucent and somewhat darkened, the radial cell closed and the anal cell open, with simplified venation. Short slender legs, the femurs dilated in the middle and tibiae straight and widened at the apex. Female metatarsi are strongly dilated (Fig. 2), particularly the first two tarsomeres which are wider than the apex of the tibiae. The male aedeagus (Fig. 3) is long and slightly arched, acuminated at the apex, the lower lamina is distinctly longer than the upper. Very simple endophalus lacking interior sclerites (Fig. 14). Short narrow tegmen (Fig. 4) with long slender acuminated parameres, bearing about six long golden apical setae. In general the morphology of the male copulatory organ differs little between species. There are only small differences in the shape of the apex of the aedeagus and in the apical setae on the parameres of the tegmen (Figs 11–13). The integument is generally testaceous yellow with a dark elytral border in some species. The males are darker and generally part of the prothorax and legs is black, whereas the legs of the females are always yellow. Brown and yellowish antennae with the last segments usually almost white.

#### Remarks.

The genus *Trypogeus* was described by Lacordaire (1869) in order to include his new species *Trypogeus albicornis*, from Malaysia. He assigned the new genus to his division “*Cohorte II. Cérambycides vrais souterrains*”, basically due to the morphology of the intercoxal protuberance on the metasternum, narrow and triangular in the males, wide and rounded in the females. The genus *Trypogeus* was included in the tribe *Apatophysides* because of its robust maxillary palpi, much longer than the labial palpi and also the weakly narrowed neck. Subsequently, with the exception of Lacordaire, the genus *Trypogeus* was considered as belonging in the subfamily Lepturinae; Boppe (1921) and Aurivillius (1912) included it in the tribe Toxotini. Nonfried (1894) described *Toxotus fuscus* from Sumatra (this was a misidentification of *Philus ophthalmicus* Pascoe), and Aurivillius (1912) later transferred it unduly to the genus *Trypogeus*. Gressitt (1951) described the genus *Paranthophylax* in order to include his new species *Paranthophylax sericeus* Gressitt from southern China and *Artelida asiatica* Matsushita, 1933 and placed that genus in the tribe Xylosteini (Lepturinae). Gressitt and Rondon (1970) also transferred *Toxotus superbus* Pic, 1922 to *Paranthophylax*. Hayashi and Villiers (1985) retained the genus *Trypogeus* in the tribe Xylosteini (Lepturinae) together with *Formosotoxotus* Hayashi, synonymized *Paranthophylax* with *Trypogeus* and placed in this genus *Toxotus aureopubens* Pic, 1903 and *Toxotus superbus* Pic, 1922 in addition to *Trypogeus javanicus* Aurivillius, 1925, and *Trypogeus apicalis* Fisher, 1936. They moved *Artelida asiatica* to the genus *Formosotoxotus*. Vives (2005 and 2007) described *Trypogeus cabigasi* from the Philippines and *Trypogeus barclayi* from Borneo, Holzschuh (2006) described *Trypogeus coarctatus* from Java, Indonesia, and Miroshnikov (2014) described two additional species, *Trypogeus murzini* and *Trypogeus gressitti*, so that currently the genus contains twelve known species. Of these, I hereby synonymize *Trypogeus apicalis* Fisher, 1936 with *Trypogeus javanicus* Aurivillius, 1925. *Trypogeus fuscus* sensu Hayashi and Villiers (1985) and Vives (2007) (nec Nonfried, 1894) should be assigned to *Trypogeus coarctatus* Holzschuh, 2006, because *Toxotus fuscus* Nonfried is a misidentification of *Philus ophthalmicus*; therefore, the genus would be reduced to ten species.

*Trypogeus albicornis* Lacordaire, 1869 W Malaysia, Java

*Trypogeus aureopubens* Pic, 1913 China (Yunnan), Thailand

*Trypogeus barclayi* Vives, 2007 Borneo

*Trypogeus cabigasi* Vives, 2005 Philippines (Mindanao)

*Trypogeus coarctatus* Holzschuh, 2006 Sumatra

*Trypogeus gressitti* Miroshnikov, 2014 Laos

*Trypogeus javanicus* Aurivillius, 1925 Java

*Trypogeus murzini* Miroshnikov, 2014 Cambodia

*Trypogeus sericeus* (Gressit, 1951) China (Fujian, Sichuan)

*Trypogeus superbus* (Pic, 1922) Laos, Vietnam

#### Keys for the identification of species

**Table:** 

1	Disc of pronotum with five gibbosities	**2**
–	Disc of pronotum with four gibbosities	**3**
2	Colour reddish, matte, clothed in brown and gold pubescence. Pronotum with a very sharp lateral spine. Female with completely yellow pronotum. Yunnan	***Trypogeus aureopubens*** (Pic)
–	Colour testaceous, with golden pubescence and slightly sharp spine. Female with completely yellowish pronotum and antennae, 16.5 mm. China	***Trypogeus sericeus*** (Gressitt)
3	Golden-yellow scutellum, sometimes blackish at the base. Head and pronotum partially black and partially yellow	**4**
–	Head, pronotum and scutellum of the male completely black. Female not known. Cambodia	***Trypogeus murzini*** Miroshnikov
4	Blackish-brown antennae with the exception of segments (9) 10 and 11 that are almost completely white	**5**
–	Antennae almost black or a testaceous brown throughout their whole length. Teguments almost black. Black scapus, labrum, clypeus, frons, elytra except for margins. Female with black pronotum and elytra black at both sides. Java	***Trypogeus javanicus* Aurivillius**
5	Elytral teguments mostly yellowish. Antennae with segments 1–6 reddish	**6**
–	Teguments almost black, clothed in golden tomentum. Antennae with all segments reddish brown except 11, which is white, 17 mm. Female not known. Sumatra	***Trypogeus coarctatus*** Holzschuh
6	Black antennae, base of segments 3–8 reddish, apical part of 9–11 white. Brown-yellow elytra, clothed in silver tomentum, sides blackish and dark spots on the disc, 15 mm. Female with entirely brownish yellow teguments, silky, 16 mm. Vietnam, Laos	***Trypogeus superbus*** (Pic)
–	Brown antennae with segments 1–2 testaceous, segments 3–8 black and 9–11 white	**7**
7	Teguments yellow, clothed in silky golden tomentum. Pronotum with 4 discal gibbosities	**8**
–	Yellowish-brown teguments, clothed in silky golden tomentum. Pronotum with yellow disc, black at both sides, 11 mm. Female with yellow legs and antennae segments 3–8 black. Malaysia	***Trypogeus albicornis* Lacordaire**
8	Elytra entirely yellow with blackish apical apex	**9**
–	Elytra completely brown with blackish sides, basal area yellowish, 12.5 mm. Female not known. Laos	***Trypogeus gressitti* Miroshnikov**
9	Pronotum with discal area yellowish and black sides, 11.5 mm. Female with yellowish pronotum. Philippines	***Trypogeus cabigasi* Vives**
–	Pronotum with discal area yellowish, anterior and posterior parts dark, 11 mm. Females completely yellowish, except for the dark elytral apex. Brunei, Borneo, Kalimantan, Sabah	***Trypogeus barclayi* Vives**

### Trypogeus albicornis

Taxon classificationAnimaliaColeopteraCerambycidae

Lacordaire, 1860

Figs 12 , 15

Trypogeus albicornis Lacordaire, 1860: 236
Trypogeus albicornis : Aurivillius 1912: 159; Boppe 1921: 44; Hayashi and Villiers 1985: 27; Vives 2007: 54; Miroshnikov 2014: 53

#### Material studied.

1 female holotype, Malaysia, Ex-Musaeo Mniszech, “TYPE” “Museum Paris coll. J.Thomson, 1952” (MNHN). 2 males, 6 females from Indonesia, Java occ., Sukabumi, 2000 m, 1893, H. Fruhstofer leg. (MNHN), 1 female from Java, Mt. Kawis, J.B. Lebdru, 1898 (ex coll. Oberthure, 1952), (MNHN). 1 male from Malaysia, Cameron Highlands, March 1987, local collector (NOC), 4 males and 1 female from the Malaysian Peninsula, Pasoh Forest reserve, Negeri Sembilan, 2–8.IV.1993, 26.III.1993, 3–9.IV.1993; 2–8.IV.1993; and 8–15.IV.1993, coarse malaise trap, K. Kimishi & K. Maeto leg. (NOC); 1 male from Malaysia, Pahang, Tanah Rata C.E., Mt. Gu. Jabar, 14–26.I.2011, T.S. Wong leg. (KMC).

#### Redescription.

Size of the male: length 9–11 mm; width 3.3 mm. Size of the female: length 14–16 mm; width 4.6 mm. General colour testaceous-yellow. Antennae with segments 1–3 yellowish, segments 4–8 brown and 9–11 white. Testaceous legs. Elytra with darkened, almost black, sutural and apical areas as well as sides including epipleura. All the body is clothed in short golden tomentum. The first sternites are testaceous-yellow, the last two are black in the males and yellow in the females. Head subquadrate, with prominent eyes. Long reddish mandibles, slightly darker in the inner margin, the external border arched and covered in long golden setae, extending from the base and almost reaching the apical part. Long slender maxillary palpi, the last segment cup shaped and truncate at apex. Labial palpi longer than the mandibles, the second segment reaching the apex. Frons with a longitudinal groove. Antennae not long, only just reaching the apical fifth of elytra in males, scapus strongly punctate and enlarged distally. Antennomere 3 long and narrow, flattened, after antennomere 4 they are slightly dilated in the external apical part. Pronotum subcylindrical, slightly wider than long (70/80) at the level of the strongly rounded lateral protuberances. Triangular scutellum with slightly rounded apex, punctate and with short golden tomentum. Long narrow elytra, rounded and very prominent humeri, narrowed at the middle and dehiscent at apex, which is rounded in each elytron. Bordered suture. Discal area with a depression from elytral base to the centre. The elytra leave the last two abdominal segments uncovered. Mesosternum and metasternum short and broad, with a very broad, subquadrate mesoepisterna, the mesocoxal cavities well separated by an angular process. Wide abdomen in females, slightly more so than the elytra, the tegument of the five visible sternitesis translucid, through which around fifty eggs can be seen in the holotype specimen, which Lacordaire (1869) probably mistook for “scattered white spots”. The sternites are finely punctate and clothed in short golden tomentum. Short robust legs, femora widened at middle, straight tibiae dilated at apex.

**Figures 15–20.**
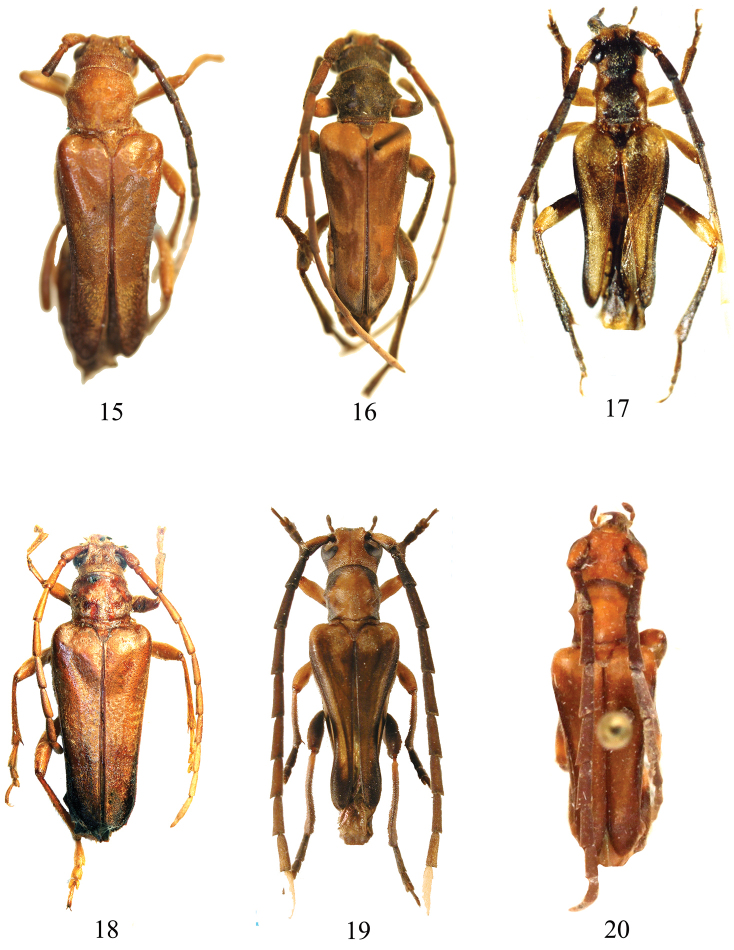
**15** *Trypogeus albicornis* Lacordaire, holotype female, Malaysia; **16** *Trypogeus aureopubens* Pic, holotype male, Yunnan **17** *Trypogeus barclayi* Vives, holotype male, Brunei **18** *Trypogeus cabigasi* Vives, holotype female, Philippines **19** *Trypogeus coarctatus* Holzschuh, holotype male, Sumatra, (Photo: L. Dembický) **20** *Trypogeus javanicus* Aurivillius, syntype male, Java.

#### Distribution.

Peninsular Malaysia, Indonesia (Java).

### Trypogeus aureopubens

Taxon classificationAnimaliaColeopteraCerambycidae

(Pic, 1913)

Fig. 16

Toxotus aureopubens Pic, 1913.
Trypogeus aureopubens : Hayashi and Villiers 1985: 27; Vives 2007: 54; Weigel et al. 2013: 68; Miroshnikov 2014: 54.

#### Material studied.

1 male holotype, “*Toxotus aureopubens* Pic, n. sp. Yunnan”,[China],P. Guerin,1924, (MNHN). 1 male from Thailand, Khao Chong, Trang, 16.IV.1969, A. Samraadkit leg. (NOC). 4 males and 1 female from Thailand, Loa Phu Ruea N.P., 13–19.IX.2006, 26.IX.2006, N. Jaroeuchai leg. (EVC); 1 male from Thailand, Thung Shlaeng mang N.P., 13–19.VIII.2006, P. Praenea leg. (EVC).

#### Redescription.

Size of the male: length 10–14 mm, width 4.3 mm. Size of the female: length 14–16 mm, width 4.5 mm. The general body colour of the male is dark brown, the base of the elytra is testaceous, antennal segments 1–3 reddish, segments 10–11 white. Legs with femora yellow-ringed at apical third and base. Elytra completely covered in a golden brown tomentum that changes direction giving it a marbled appearance. The male has a short broad head, maxillary palpi as long as mandibles, the third segment two times as long as wide and truncate at the apex with a slight depression at each side. Antennae reaching past apex of elytra after the ninth segment. Vertex of head slightly furrowed between antennal bases. Almost smooth gular area with transverse grooves. Subquadrate pronotum, the maximum width is in the front third at the height of the lateral calli that are protruding and sharp, with a slightly convex area at the front. Disc of pronotum with five rounded and well defined gibbosities. Posterior border of pronotum weakly sinuate. Prosternum finely punctate with large prominent procoxae, prosternal process very fine and narrow. Triangular scutellum widely rounded at apex, finely punctate and with long golden tomentum. Long elytra, weakly narrowed at the middle with prominent rounded humeri. Base with a depression at either side and somewhat raised around the scutellum. Fine and dark sutural border. Elytral apex weakly rounded and weakly dehiscent sutural angle. Finely punctuate mesosternum with separated mesocoxae and weakly projecting coxae. Metasternum with large subquadrate episternae, the coxae almost joined in the male. The abdomen has five visible sternites, these are dark but have a yellowish posterior margin. The entire underside of the body bears long silver setae. Short robust legs with femora dilated at the middle and presenting a smooth, shiny oval area at the distal part. Tibiae straight and widened at apex. Short narrow tarsi, the first segment of the metatarsi slightly longer than the remaining tarsi together.

#### Distribution.

China (Yunnan), Thailand.

### Trypogeus barclayi

Taxon classificationAnimaliaColeopteraCerambycidae

Vives, 2007

Fig. 17

Trypogeus barclayi Vives, 2007: 54.
Trypogeus barclayi : Miroshnikov 2014: 59.

#### Material studied.

1 male holotype from Brunei, II-III.1992, (E 115, 7° - N 4, 34°) Kuala Belalong FSC, N. Mawdsley leg. (BMNH); 1 male Paratype, *idem*, (EVC); 1 female from Malaysia SW, Sabah, Long Pa Sia, Payakalaba, 1010 m, 13–13.IV.1987, C.V. Achterberg leg. (EVC); 1 male from Malaysia SW, Sabah, Borneo, Headquarters, 5.V.1980, A. Sakai leg. (NOC); 3 males from Indonesia, Kalimantan, Timur, Bikit Bangkiria, 14–20.IX.1999, 8–14.XI.2000, 11–17.X.2000, H. Makihara leg. (NOC); 1 male from Indonesia, east Kalimantan, Bukit Soeharto, 11–18.X.1996, H. Makihara & H. Kinumura leg. (NOC); 1 male from Malaysia SW, Sabah, Trus Madi, V.2000 (NOC).

#### Redescription.

Size of the male 9–11 mm long and 3.9–3.2 mm wide. Body completely testaceous except for the external margin of mandibles, the lateral margin and apex of elytra, the first seven antennal segments and the tibiae, which are almost black. The occiput and thoracic disc have a large brown-black spot. The underside of the body is brown except for the abdominal sternites which are almost black. Broad short head, prominent eyes with medium facets. The lower lobe is almost double the length of the upper lobe. The mandibles are broad, arched and protruding; the inner margin is smooth, lacking a medial tooth. The antennae are long and robust, extending past elytral apex from the third antennomere, the last four antennomeres are yellowish. Pronotum subquadrate, slightly longer than wide with four discal protuberances, one at each side of the pronotal middle. A large brown spot extends longitudinally from the front to the rear border, narrowing at apical third. The entire pronotal surface is coarsely punctate, almost rugged. The hind margin is doubly sinuate. Yellowish prosternum with the front and rear parts brown. Prosternal process long and narrow, the coxal cavities opened behind and procoxae extended at an angle to the sides. Dark brown triangular scutellum with a central depression. The central plate of the mesonotum lacking a stridulatory area. Subconical elytra, wider than pronotum with humeri very obtuse, rounded and projecting upwards. Bordered protruding suture. Apex of elytra rounded and dehiscent. The elytra are narrowed after the middle, widening slightly at apical fifth. Elytral punctation is coarse and granulose, decreasing after apical third. The entire elytral surface is clothed in golden tomentum that becomes brown at sides and brown-black at apex. Legs short and robust, tibiae widened at apex. Clothed in golden tomentum except for tibiae that are covered in dense blackish pubescence. Tarsi not widened and with the first segment of mesotarsi and metatarsi longer than the second and third together. The lower part of the body is finely punctate and clothed in golden tomentum. Copulatory organ of male elongate (Fig. 13) slightly arched with a strongly acuminate apex and the upper lamina narrower and thinner than the lower, not reaching the apex. Lateral parameres long and slender, bearing long setae at apex and some short setae on the inner side.

This species is the smallest in the genus, it can be distinguished from the others by the almost black spots on head and pronotum and the tibiae with black pubescence. The most similar species is *Trypogeus javanicus* Aurivillius, from which it can be separated by the characters mentioned, by the different colouration of elytra and the morphology of the pronotum.

#### Distribution.

Borneo, including Sabah, Brunei, and Kalimantan.

### Trypogeus cabigasi

Taxon classificationAnimaliaColeopteraCerambycidae

Vives, 2005

Fig. 18

Trypogeus cabigasi Vives, 2005: 303.
Trypogeus cabigasi : Miroshnikov 2014: 59.

#### Material studied.

1 female holotype from Philippines, Mindanao, Bukidnon, Impasung-ong,10.V.2002, E. Cabigasi leg. (EVC); 2 males from Philippines, Mindanao, Mt. Apo, 5.VII.1978 and 5.VIII.1978, O Yata leg. (NOC); 1 female from Philippines, Mindanao, Mt. Apo, 26.30.III.1980, T. Hirowatari & Y. Funatsa leg. (NOC); 1 female from Philippines, Mindanao Is., N Agusan, V-VI.1977, R.M. Lumawig leg. (KMC).

#### Redescription.

Size of the male: 13–15 mm; width 3.2–4.5 mm. Size of the female: 16.5 mm. Entirely testaceous except for the internal margin of mandibles, the apex of labial palpi, the anterior margin of pronotum, half of the elytral margin and the elytral apex, which are dark brown. Antennae reddish except for the last three segments, that are yellowish white. Underside of body testaceous, lacking the yellow markings on the abdomen present in the other species of the genus. Broad short head with large broad long mandibles, presenting a longitudinal furrow on their external margin and almost completely covered with golden pubescence. Small and slightly convex eyes, small-faceted. Dorsal surface of head furrowed by a longitudinal impression between the antennal bases. Broad neck, not narrowing behind the eyes. Antennae slightly longer than elytra; third segment clearly longer than fourth. All antennomeres elongated and cylindrical, except for segments 9 to 11, fairly flattened and yellowish. Eleventh antennomere appendiculate. Rather square pronotum, almost as long as wide, with a small protuberance on each side, slightly in front of the middle. Pronotal disc with four inconspicuous protuberances, the two anterior closer together than the two behind. Entire surface of pronotum and head strongly punctate, almost rugged, both covered by a short golden tomentum that becomes longer at the sides. Triangular scutellum covered by dark pubescence. Elytra relatively short, wider at the base and tapering apically, not narrowing behind the apical quarter. Round protruding humeri; suture narrowly bordered and raised. Basal third of the elytra with an oblique depression running from the humerus to the suture, delimiting a convex area. Elytral punctation coarse and scattered, formed by black disseminated punctures not reaching the posterior half of the elytra. Round dehiscent elytral apex. Elytra covered by short golden and silky tomentum which becomes reddish or black at the apex and margin of the apical third. Legs short and robust, covered by long silky golden pubescence. Tibiae slightly dilated apically. Tarsi of males not enlarged; the first metatarsal segment longer than the second and third together. Long narrow onychium. Underside of the body yellowish-testaceous and covered with long golden silky tomentum. Aedeagus of male (Fig. 11) slightly arched with a strongly acuminate apex. Lateral parameres long and slender bearing long setae at apex and some short setae on interior side.

#### Distribution.

Philippines (Mindanao).

### Trypogeus coarctatus

Taxon classificationAnimaliaColeopteraCerambycidae

Holzschuh, 2006

Fig. 19

Trypogeus fuscus : auct. nec *Toxotus fuscus* Nonfried, 1894: 209.
Trypogeus coarctatus Holzschuh, 2006: 207.

#### Material studied.

1 male from Indonesia, Bandar Baru, Sumatra Utara, 3-V-1999, S. Tsuyuki leg. (NOC,); 1 male from Indonesia, N Sumatra, Brastagi, 27.IV-4.V-1988, A. Saito leg. (*in* NOC); 1 female from Indonesia, SW Sumatra, Marang, W. Doherty leg, 1890 (ex coll. Oberthur) (MNHN); 1 female from Indonesia, W Sumatra, Merapi, IV.1991, S. Ymada leg. (KMC).

#### Redescription.

Size of the male: length 10–12 mm; width 3.6 mm. Size of the female: length 14–17 mm; width 4.2 mm. The general colour of the integument is testaceous yellow, males are darker and browner. Head brown except for the upper part which is yellowish. The antennae of males are completely brown, except for the last antennomere which is yellow; in females the scapus is testaceous, the antennomeres 3–8 are brown and the remaining 9–11 are yellow. The legs of the males are brown except for the femora which are mostly yellow. The legs of the females are completely yellowish. Pronotum brown in males with the discal area yellowish, in females it is almost completely testaceous. Entirely brown elytra, except for the basal area which is yellower, slightly lighter in the females. Elytra wider than pronotum with very obtuse humeri, rounded and projecting upwards. The suture is bordered and protruding. Apex of elytra rounded and dehiscent. The elytra are narrowed after the middle, widening slightly at apical fifth. Underside of the body and head brown except gular area which is yellow. Brown epimerae and abdominal sternites yellowish in both sexes. Large head with very short broad neck. The mandibles are long and almost entirely covered in golden tomentum. Translucid epistome, rectangular labrum bearing long golden setae on the free margin. Maxillary palpi longer than mandibles, with the last segment fusiform. Labial palpi shorter with the last segment cup-shaped. Base of the antennae with a granulous crest that extends to base of mandibles. Large prominent eyes, medium granulation. Posterior part of the head with dense golden tomentum. Antennae of males slightly longer than elytra, in females only reaching apical third. Long slender antennomeres, slightly flattened and angulose at the external distal part from antennomeres 4–10, the last is fusiform. Cylindrical pronotum, almost as wide as long in males, transverse in females, weakly arched sides with a barely protruding gibbosity before the middle. Discal area with four rather indistinct protuberances, the two anterior ones are very close to each other, the posterior pair slightly transverse in the males. Anterior border of pronotum finely margined, the posterior border is sinuate with double margination. Narrow prosternum, prosternal process in the shape of a fine lamina, hardly dilated behind. Widely opened procoxal cavities. Conical prominent coxae contiguous. Mesocoxae slightly separated in males and much more so in females, the metacoxae, contiguous in males, are separated in the females. Triangular scutellum rounded at apex. Elytral apex round and dehiscent. Elytra covered by golden tomentum which becomes reddish or black at the apex and margin of the apical third. Legs short and robust, covered by long silky golden pubescence. Tibiae slightly dilated apically. The male aedeagus (Fig. 14) is long and slightly arched, acuminated at the apex, the lower lamina is distinctlylonger than the upper. Very simple endophalus lacking interior sclerites.

#### Remarks.

Two syntype specimens described as *Toxotus fuscus* Nonfried, 1894, were examined that correspond in reality to *Philus ophthalmicus* Pascoe, 1886 (Fig. 6). It is hereafter established that the species assigned as *Trypogeus fuscus auct.* belong to *Trypogeus coarctatus* Holzschuh. A lectotype is designated between the two syntypes studied by Nonfried, in order to establish the exact taxonomic status of the species. Lectotype, 1 male from NW Sumatra, Tebing Tinggi, Dr. Schultheiss; 12.V.1884, (DEI) (Figs 7–8).

#### Distribution.

Indonesia (Sumatra).

### Trypogeus gressitti

Taxon classificationAnimaliaColeopteraCerambycidae

Miroshnikov, 2014

Fig. 26

Trypogeus gressitti Miroshnikov, 2014: 55.
Paranthophylax superbus Gressitt & Rondon, 1970: 31 (partim).
Trypogeus superbus : Hayashi and Villiers 1985: 4, 29 (partim); Vives 2007: 54 (partim).

#### Material studied.

1 male holotype, Laos, Phou Kou Khouei, 1.X.1963, Vientiane, ex. coll. J.A. Rondon (BPBM); 1 female paratype, Laos, Tampheng, 15.X.1965, ex coll. J.A. Rondon (BPBM).

#### Redescription.

Size of the male: length 10–12 mm; width 3.6 mm. Size of the female: 15.3 mm. Dorsal part of head entirely or only partially red-yellow, particularly behind eyes and in area of clypeus, black-brown between eyes and on vertex, sides and underside mostly black and dark brown with a lighter gula; black eyes and mandibles; red-yellow scutellum; elytra almost completely covered by a contrasting red-yellow fascia at base with a complicated pattern, lateral margin partly black-brown, remaining surface red-yellow. Prosternum entirely dark brown with a reddish hue, as well as mesosternum completely or mostly;part of the metasternum always including episternae and adjoining lateral surface; sternites either completely or excluding both the apical part of penultimate sternite and the last sternite, as well as last tergite; remaining surface yellow. Head narrower than pronotum at level of lateral tubercles; dorsally mostly with a granulate-rugose sculpture, with coarse confluent punctation covering most of the vertex and a sharp longitudinal median groove. Moderately developed antennal tubercles. Ventral side between eyes with abundant coarse bumps; conspicuous transverse wrinkles on gula; sharp, irregular, partly condensed punctationon either side. Antennae much longer than body, reaching beyond apex of elytra. Pronotum at level of lateral tubercles as wide as at base and as long, lateral tubercles clearly more strongly developed in females than in the males; structure of tubercles on disc and impression before tubercles at base the same as in males, sharp at apex, slightly sinuatelateral margin narrowed at apex. Disc with four tubercles, two at the base and a further two in the middle, with a very sharp, transverse, oblique impression before tubercles at base; fine granular sculpture. Triangular scutellum, subequal in length and width at base. Elytra strongly narrowed towards apex; sides straighter in apical third; conspicuously diverging along suture at apex. Prosternal process very narrow between coxae; moderately wide mesosternal process; metasternum with fine, dense, coarse punctation; wide metepisterna, moderately narrowed towards apex; sternites with fine, dense, coarse punctation. Legs robust, moderately long; femora thickened, but not claviform; metatibiae clearly emarginate at apex. Well-developed decumbent pubescence that lies in different directions giving elytra an iridescent appearance, common to other species in the genus.

Female. Head red-yellow, except for the strongly darkened apical part of mandibles and the margin of genae; entire pronotum, venter, legs, antennomeres 1–2; elytra except for the darkened apex and partially black margin. Antennomeres 3–8 black, except for a red-yellow base, most noticeably antennomeres 3 and 4 (both dorsally and ventrally); the two or three last antennomeres (see below) usually entirely pale. Head considerably narrower than pronotum at level of lateral tubercles; antennal tubercles more strongly developed and sharper than in the male; sculpture about the same as in the male; antennae usually slightly longer than body, reaching beyond apex of elytra.

**Figures 21–26.**
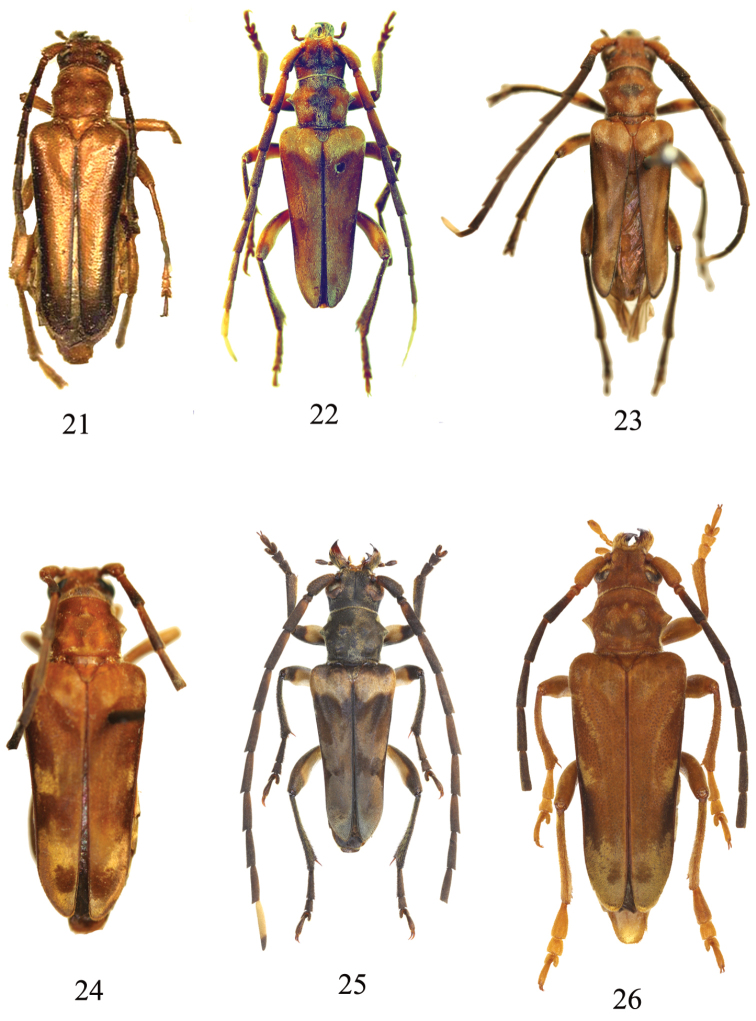
**21** *Trypogeus apicalis* Fisher, holotype female, Java (=*Trypogeus javanicus*) **22** *Trypogeussericeus* (Gressitt), neotype, China **23** *Trypogeus superbus* (Pic), holotype, Tonkin **24** Trypogeus superbus v. innotatus Pic, holotype, Vietnam (=*Trypogeus superbus* S. str.) **25** *Trypogeus murzini* Miroshnikov, holotype male, Cambodia **26** *Trypogeus gressitti* Miroshnikov, holotype male.

#### Distribution.

Laos.

### Trypogeus javanicus

Taxon classificationAnimaliaColeopteraCerambycidae

Aurivillius, 1925

Fig. 20

Trypogeus javanicus Aurivillius, 1925: 2
Trypogeus apicalis Fisher, 1936: 171. **syn. n.**
Trypogeus javanicus : Hayashi and Villiers 1985: 27; Makihara et al. 2002: 190; Vives 2007: 54.
Trypogeus apicalis : Hayashi and Villiers 1985: 27; Vives 2007: 54.

#### Material studied.

Syntype 1 male (Nº23248); Syntypes, 1 female (nº 2350) Java, Tengger Berg, 4.000 f.h., Fruhstorfer leg. (NRSC); 1 male (Nº 2349), 1 female (Nº.2351). Holotype of *Trypogeus apicalis* Fisher, 1936, from Indonesia; 1 female, Java, G. Tangkoeban 4000–5000 Voet Prahoe Preanger, 16.II-8.III.1933, Drescher, F.C., (USNM); 1 male from Indonesia, Java, Mt. Djampang, local collector (EVC); 1 male from Java, Pengalenhan, 4000, 1893, H. Fruhstorfer leg. (EVC); 1 female from W Java., Toegoe, local collector (EVC); 1 female from Java, Preangar, P.F. Sijthaff (EVC); 1 male from Java, ex coll.Moffarts (IRSNB); 1 female from Malang, Java (IRSNB).

#### Redescription.

Size of the male: length 9–11 mm; width 3.6 mm. Size of the female: length 14–16 mm; width 3.8 mm. The male is dark and clothed in pale tomentum. Yellowish labrum, clypeus, scapus, elytra, fore-and mid-femora. Yellow pronotum and abdomen. Frons naked, wrinkled and punctate. Antennae as long as the body with segments 3–5 subequal, segments 4–10 with slightly serrated external margin. Eyes with fine granulation, weakly emarginated. Pronotum clothed in dense white tomentum, shape subcylindrical, the widest part is before the middle, the sides have two small lateral tubercles, discal area with four gibbosities. Triangular scutellum with rounded apex and dense white silky tomentum. Elytra with broad base, almost twice as wide as the base of the pronotum, acuminate at apex with emarginate sides, each elytron is individually rounded and weakly dehiscent. Discal area with a depression from elytral base to the middle. Bordered suture. Basal tomentum white and silky, darkened suture. Anterior coxae subcontiguous, midcoxae rather separate, mesosternal process with wide, strongly bordered apex. Anterior femora thickened after apical half, longer than fourth abdominal segment. Female testaceous yellow, with golden-yellow tomentum on the head, very abundant on the external side of mandibles. Antennomeres 3–8, lateral margin and apex of elytra dark, almost black. Antennomeres 1–3 testaceous, 9–11 white. Pronotal disc with four inconspicuous gibbosities.

#### Remarks.

I have studied the holotype of *Trypogeus apicalis* Fisher, 1933 (Lingafelter et al. 2014: 18) and can hereby confirm that it corresponds perfectly to the female of *Trypogeus javanicus*, which is why its synonymy with *Trypogeus javanicus* Aurivillius, 1925 is proposed.

#### Distribution.

Indonesia (Java).

### Trypogeus murzini

Taxon classificationAnimaliaColeopteraCerambycidae

Miroshnikov, 2014

Fig. 25

Trypogeus murzini Miroshnikov, 2014: 57.

#### Material studied.

1 male holotype from Cambodia, Phuom Bokor Nat. Reserv., 600 m, 24–28.XI.2007, S. Murzin leg. (SMC).

#### Redescription.

Size of the male: length 11.3 mm; width 3.3 mm. Head almost completely black, with only small, unequally developed, yellow specks behind antennal tubercles; eyes and mouthparts partly lighter. Antennae on dorsal side almost entirely black, bases of antennomeres 1 and 3–8 reddish, last antennomere beige, except for its black apex; ventral side of antennae mostly beige, apices of antennomeres 1, 3–8 and last black, antennomeres 9 and 10 entirely black, antennomere 2 almostcompletely black. Entirely black pronotum and scutellum. Black elytra, partly black-brown at the very base before a wide yellow fascia with zigzag margins, and then it is black-brown until the apex. Venter with almost entirely black pro- and mesosterna, as well as sternites, a partly black metasternum. Legs black except for the yellow coxae. Head clearly narrower than pronotum at level of lateral tubercles; dorsally mostly with a clearly coarsely granulated sculpture, vertex predominantly with a coarse confluent punctuation. Long mandibles, strongly curved right mandible, ventral side between eyes coarsely and abundantly tuberculate; with an inconspicuous sculpture; at either side there is a clear, sparse, but in places condensed punctuation. Antennae much longer than body, reaching beyond apex of elytra by antennomere 8. Pronotum with lateral tubercles very well-developed, sharp at apex, lateral margin moderately narrowed towards its base; disc with four moderately conspicuous gibbosities, two at base and a further two in the middle; finely granulose sculpture and fine punctation. Barely elongate, triangular scutellum. Elytra strongly narrowed towards apex; straighter in apical third; each elytron narrowly rounded at apex. Very narrow prosternal process between coxae; moderately wide mesosternal process; metasternum with dense, coarse punctation; wide metepisterna, moderately narrowed towards apex. Sternites with fine, dense, rugulose punctation; last sternite slightly impressed, broad but slightly emarginate at apex. Legs robust, moderately long; femora thickened, but not claviform; metatibiae clearly emarginate at apex.

#### Distribution.

Cambodia.

### Trypogeus sericeus

Taxon classificationAnimaliaColeopteraCerambycidae

(Gressitt, 1951)

Fig. 22

Paranthophylax sericeus Gressitt, 1951: 50.
Trypogeus sericeus : Hayashi and Villiers 1985: 27; Makihara 1999: 48; Chiang and Chen 2001: 37; Hua 2002: 235; Vives 2007: 54; Hua et al. 2008: 284; Miroshnikov 2014: 53.

#### Material studied.

1 male neotype (designated here) 12.2 mm long and 4.3 mm wide, originating from China, Fujian Prov., Chong’an, Xingcun, Sangang, alt. 720–850m: 1960.VIII.10, leg. Shengqiao Jiang (IZAS coll., 1858382) with red label of neotype; 1 male from China, Sichuan, Huili, Yimen, alt. 2000–2200 m, 30.VII.1974, leg. Yinheng Han, IZAS coll., 1858383;1 female from China, Fujian, Sangang, 20.IX.1979, leg. Jinxing Gong, IZAS coll.1858381. A neotype is designated here because the original type described by Gressitt (1951) has not been found in the collection of Fujian Agriculture and Forestry University.

#### Redescription.

Size of the male, length 10.6–14.1 mm; width 3.5–4.6 mm. Size of the female16.4 mm. Overall colour of the male testaceous-yellow, clothed with golden pubescence. Head subquadrate, longitudinally furrowed, eyes not very prominent. Mandibles reddish on the inner margin and blackish on external side and apex. Generally with a blackish spot between the eyes, in some specimens it can be completely black. Antennae long, extending past apex of elytra at antennomere 9, slightly serrated after antennomere 4, usually testaceousbrown from the scapus to the fourth antennomere, the last two segments are usually yellowish. Pronotum subquadrate (23/30) with four protuberances in the centre of the disc and a fifth in the middle of the posterior border. Prominent lateral gibbosities barely sharp. Disc testaceous red with a blackish cross-shaped spot. Prosternum short with very narrow prosternal process slightly enlarged towards the rear. Procoxal cavities opened, narrow mesosternum with coxal cavities separated by a bilobed and strongly punctate mesosternal process. Scutellum black and rounded at apex. Elytra long and slightly narrowed after basal third, rounded humeri very prominent. Finely bordered elytral suture slightly darker than the rest of the elytra. Elytra hunched at base, behind scutellum, with strong sparse punctures; the rest of the elytra are reddish-brown with a darker epipleural margin and much finer and sparser punctation. Apex of each elytron rounded, dehiscent sutural angle. Abdominal segments brown and punctate, clothed in golden pubescence. Short robust legs with femora dilated at middle, testaceous except for the apex which is brown. Straight tibiae vey dilated at apex, usually brown. Tarsi short and narrow, except for the first metatarsus which is almost as long as the others together. Aedeagus long and slender, very narrow median lobe, upper apical lamina shorter and narrower than the lower. Smooth endophalus lacking chitinous sclerites. Short narrow lateral parameres with rounded apex and bearing some long golden setae (Fig. 11).

#### Distribution.

China.

### Trypogeus superbus

Taxon classificationAnimaliaColeopteraCerambycidae

(Pic, 1922)

Fig. 23

Toxotus superbus Pic, 1922: 22.
Toxotus superbus var. innotatus Pic: 16. **syn. n.**
Paranthophylax superbus : Gressitt and Rondon 1970: 31 (partim).
Trypogeus superbus , Hayashi and Villiers 1985: 27 (partim); Vives 2007: 54 (partim); Miroshnikov 2014: 54.

#### Material studied.

1 male holotype, Hao Djing, Tonkin, “Type”, “Toxotus superbus”; “Museum Paris, coll. M.Pic” (MNHN). 1 female from W Tonkin, region of Hoa Binh, R.P.A. de Cooman, 1918, (ex coll. Oberthur, 1952), (MNHN). 1 female holotype of *Toxotus superbus v. innotatus* Pic, from Vietnam, Hoa Bihn (MNHN); 1 female from Vietnam, Lukbiho, Tonkin, (MNHN); 1 male from Laos, Kinsey Kuany, (MNHN); 1 female from South Vietnam, Bao Loc, 1800 m, 25.IV.1993, Sinaiev & Simonov leg. (EVC).

#### Redescription.

Size of the male: length 9–13 mm; width 4 mm. Size of the female: length 15.8 mm. Males generally have testaceous-yellow teguments; the mandibles, antennomeres 3–9, tibiae and tarsi are brown, almost black. The front and rear margins of the pronotum are brown, as well as a rounded spot in the centre of the disc. Reddish palpi. Underside of head, prosternum, sides of mesosternum and metasternum and abdominal tergites brown. Base of antennomeres 1–4 yellowish, antennomere 10 half yellow and the other half testaceous, antennomere 11 yellow. Abdominal segments brown in males and yellow in females. Head large, almost transverse, the neck strongly sunk into the pronotum, eyes small and not very prominent. Mandibles strongly curved at apex, the right mandible crosses over the left. Long palpi, the last segment is oval and truncate with a depression on each side. Long antennae, extending past apex of elytra after antennomere 9, somewhat dentate externally. Head grooved longitudinally and strongly punctate. Pronotum subcylindrical, the maximum width is at the height of the lateral protuberances which are small, conical and sharp. The discal area has four gibbosities and is more strongly punctate in the centre where it has a dark spot. Long golden tomentum. Short broad prosternum. Very slender intercoxal process, barely widened behind. Procoxal cavities opened and very extended to the sides, reaching sides of pronotum. Triangular scutellum with rounded apex. Elytra narrow with rounded prominent humeri, slightly narrowed at the middle and slightly rounded at apex. Suture blackish and finely bordered, sides and epipleurae also blackish. Discal area slightly raised and convex. Mesosternum with short broad mesoepisternae, finely punctate and pubescent. Mesocoxal cavities open laterally. Broad mesosternal process and coxae very separate. Metasternum with coxae almost in contact. Abdomen with short wide sternites, finely punctate and with golden tomentum. Legs short and robust with femora widened at middle. Tibiae straight and dilated at apex. Tarsi dark and punctate, the first segment of metatarsi is longer than the other segments together.

#### Distribution.

Laos and Vietnam.

## Supplementary Material

XML Treatment for Dorcasominae

XML Treatment for Trypogeus

XML Treatment for Trypogeus albicornis

XML Treatment for Trypogeus aureopubens

XML Treatment for Trypogeus barclayi

XML Treatment for Trypogeus cabigasi

XML Treatment for Trypogeus coarctatus

XML Treatment for Trypogeus gressitti

XML Treatment for Trypogeus javanicus

XML Treatment for Trypogeus murzini

XML Treatment for Trypogeus sericeus

XML Treatment for Trypogeus superbus
